# The lasting impact of COVID-19 on surgical training from the perspective of surgical residents and consultants in Saudi Arabia: a nationwide cross-sectional study

**DOI:** 10.1186/s12909-023-04302-4

**Published:** 2023-05-11

**Authors:** Jumanah T. Qedair, Wejdan A. Alnahdi, Hatan Mortada, Abdulrahman A. Alnamlah, Raghad Z. Almadani, Alqassem Y. Hakami

**Affiliations:** 1 College of Medicine, King Saud bin Abdulaziz University for Health Sciences, Jeddah, Saudi Arabia; 2 King Abdullah International Medical Research Center (KAIMRC), Jeddah, Saudi Arabia; 3 Division of Plastic Surgery, Department of Surgery, College of Medicine, King Saud University, Riyadh, Saudi Arabia

**Keywords:** COVID-19, Surgical education, Training, Surgery, Saudi Arabia

## Abstract

**Background:**

Since the start of the COVID-19 pandemic, many precautionary measures have been set to curb the transmission of the virus. That has led to changes, most notably in surgical education, like lack of surgical exposure and clinical activities. However, the question aiming at the impact of changes made by the COVID-19 pandemic on surgical education and its extent remains unanswered.

**Materials & methods:**

An electronic survey was distributed among surgical residents and consultants from all over Saudi Arabia, starting from the 6th till the 21st of July, 2021. Descriptive statistics were presented using counts and proportions (%). Study subjects were compared with the different perspectives during the COVID-19 pandemic by using Chi-square test. A p-value cut-off point of 0.05 at 95% CI was used to determine statistical significance.

**Results:**

A total of 243 out of 500 surgical residents and consultants responded to the survey, giving a response rate of 48.6%. The majority were general surgeons (50.5%) and cardiothoracic surgeons (21.8%). Nearly 66% of surgeons, both residents and consultants, strongly agreed on the importance of training for infectious disease outbreaks. 44.7% of the consultants and 48% of the residents showed their willingness to respond to the pandemic regardless of its severity. Over 70% of surgeons agreed that developing clinical skills was compromised by the COVID-19 pandemic, and 40% expected a negative impact of the COVID-19 on their operative skills. Simulation was ranked best for disaster medicine training by over 77% of the respondents. The most common concern among surgeons during the COVID-19 pandemic was their family’s health and safety. Regarding virtual curriculum components, online practice questions and surgical videos were preferred by the surgical consultant and resident, respectively.

**Conclusions:**

Although the COVID-19 pandemic has impacted surgical education, it has highlighted the alarming need for adopting new components. For surgical training programs, we recommend improving the virtual curriculum, incorporating disaster medicine training, providing psychological services, and prioritizing immunization and treatment access for surgeons’ families.

**Supplementary Information:**

The online version contains supplementary material available at 10.1186/s12909-023-04302-4.

## Introduction

On the 11th of March 2020, the World Health Organization (WHO) officially announced that COVID-19 is a global pandemic that requires strict precautionary measures across the globe. Since the discovery of the first confirmed case of COVID-19 in Saudi Arabia, on the 2nd of March 2020, the number of recorded cases has increased. [1] COVID-19 is mostly associated with fever, dry cough, and fatigue. [2]

Like many other systems in modern life, healthcare system and medical education have been disrupted with no available practical alternatives. Medical education has been affected specifically by the pandemic, and this effect has been established in the literature. [3, 4] COVID-19 can noticeably impact the medical education and training due to the precautionary measures and restrictions imposed by many countries. For instance, the reduced exposure to, postponement, or cancellation of in-person clinical sessions has negatively impacted resident physicians. Decreased hours spent on studying and performing routine clinical duties are examples of COVID-19 impact. [5] As a result of removing all forms of direct contact with patients, medical education has depended mostly on e-learning, which can include online lectures and quizzes and utilisation of educational software to facilitate the learning process. Although e-learning can foster self-learning, losing the contact with patients is of a great concern. [6]

A study done by Z.V. Fong et al., found that residents’ hands-on skills can be significantly affected due to the reduced exposure to elective surgery cases. [7] Importantly, during the COVID-19 pandemic, many countries have restricted the surgeries and allowed only urgent operations to be performed, whereas elective surgeries have been postponed resulting in less available learning experiences. In Saudi Arabia, surgical interventions in ministry of health’s hospitals in 2019, prior to the pandemic, exceeded 500,000 surgeries from a wide spectrum of surgical specialties in comparison to 2020, when they dropped to approximately 400,000 surgeries. [8] Medical teams have also drastically decreased in size, leading to much less contact with patients in addition to surgical experiences. Moreover, many outpatient clinics have been reduced, or moved online, reducing patient exposure to trainees. Replacing bedside teaching and regular clinical instruction with online alternatives has also been noticed. [9].

The COVID-19 pandemic has clearly impacted the surgical education and training. Although this topic has been covered in a number of recent studies, no local studies have assessed the perspectives of surgical residents and consultants regarding how the pandemic has affected their training program in Saudi Arabia. Thus, the purpose of this study was to evaluate the impact of the COVID-19 pandemic on the local surgical training from the perceptions of surgical residents and consultants.

## Methods

The questionnaire of this quantitative cross-sectional study was structured based on the available literature with similar objectives, which was previously approved to be validated and internally consistent [10]. Two specialists in the field of surgery and medical education were invited to critically revise the survey. Subjects were included if they were surgical residents or consultants working at a primary, secondary, tertiary hospital. Any surgical residents or consultants not working in Saudi Arabia were excluded. Also, incomplete surveys were not included.

The items of the questionnaire targeted surgical residents and consultants working in Saudi Arabia to evaluate their perception of COVID-19 footprint on surgical education; all statistics regarding surgeons in this paper concerned both residents and consultants. In addition to questions targeting demographics, questions aiming at evaluating COVID-19 impact included program preparedness, response to the pandemic, COVID-19 impact on the program, and disaster medicine training. Moreover, respondents were questioned on their virtual curriculum components (the ones became part of e-learning after the pandemic) and concerns during the pandemic. A combination of binary (yes/no), multiple choice, ranking questions, and statements with 5-point Likert scales showing the extent of agreement was applied to this questionnaire.

The study was conducted under the approval from King Abdullah International Medical Research Center (KAIMRC) institutional review board (Study Number #SP21J/370/07). The self-administered survey was distributed online via social media applications, mainly WhatsApp, to a randomly selected 500 surgical residents and consultants working at different primary, secondary, and tertiary hospitals from all regions of Saudi Arabia. Data were collected for 15 days, starting from the 6th till 21st of July 2021, after introducing COVID-19 vaccines and starting the nationwide vaccination campaigns.

### Statistical analysis

After data cleaning and transformation from Excel format (Microsoft) to Statistical Packages for Software Sciences (SPSS) (26th version for Microsoft; IBM, Armonk, N.Y.), descriptive statistics had been presented using counts and proportions (%). Mean and standard deviation were calculated for each question. Study subjects were compared with the different perspectives during the COVID-19 pandemic by using Chi-square square test. A p-value cut-off point of 0.05 at 95% CI was used to determine statistical significance.

## Results

### Socio-demographic characteristics

A total of 243 out of the randomly selected 500 surgical residents and consultants responded to the survey, resulting in a response rate of 48.6%. Most of the surgical consultants were older than 35 years old, while most of the surgical residents aged between 25 and 35 years old (p < 0.001). The majority of the respondents were males, accounting for 53.2% and 55.1% of the surgical consultants and residents, respectively. 72.3% of the consultants were married, while 68.9% of the residents were single (p < 0.001). Over 95% of the participants were Saudi, and the Western region constituted most of the responses. Working for 5 years or less was more common among residents (p < 0.001). More details are shown in Table 1.

**Table 1:** Socio-demographic characteristics of consultant and resident surgeons ^(n=243)^

Study data	Surgeon		P-value ^§^
	Consultants N (%) (n = 47)	Residents N (%) (n = 196)	
Age group			
• < 25 years	02 (04.3%)	52 (26.5%)	
• 25–35 years	10 (21.3%)	135 (68.9%)	< 0.001 **
• > 35 years	35 (74.5%)	09 (04.6%)	
Gender			
• Male	25 (53.2%)	108 (55.1%)	0.813
• Female	22 (46.8%)	88 (44.9%)	
Nationality			
• Saudi	45 (95.7%)	189 (96.4%)	0.824
• Non-Saudi	02 (04.3%)	07 (03.6%)	
Marital status			
• Prefer not to answer	05 (10.6%)	05 (02.6%)	
• Single	04 (08.5%)	135 (68.9%)	< 0.001 **
• Married	34 (72.3%)	51 (26.0%)	
• Divorced	04 (08.5%)	05 (02.6%)	
Region of residence			
• Central region	10 (21.3%)	27 (13.8%)	
• Eastern region	12 (25.5%)	41 (20.9%)	
• Western region	19 (40.4%)	61 (31.1%)	0.069
• Northern region	04 (08.5%)	37 (18.9%)	
• Southern region	02 (04.3%)	30 (15.3%)	
Working hospital			
• Primary hospital	08 (17.0%)	18 (09.2%)	
• Secondary hospital	21 (44.7%)	81 (41.3%)	0.194
• Tertiary hospital	18 (38.3%)	97 (49.5%)	
Years of working as a physician			
• ≤ 5 years	02 (04.3%)	158 (80.6%)	
• 6–10 years	18 (38.3%)	27 (13.8%)	< 0.001 **
• > 10 years	27 (57.4%)	11 (05.6%)	

^§^ P-value has been calculated using Chi-square test

** Significant at p < 0.05 level

**Fig. 1:**
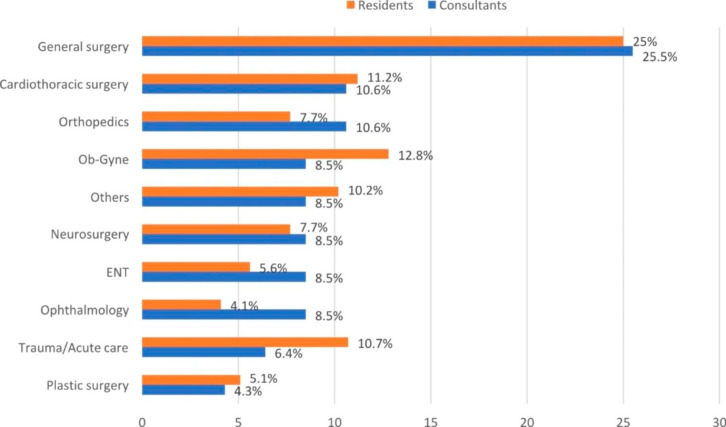
Distribution of surgical specialty

In Fig. 1, the study outcomes showed that a quarter of each of residents and consultants were general surgeons, while 11.2% and 10.6% of residents and consultants were cardiothoracic surgeons, respectively. 

### Surgeons’ perspective on pandemic preparation

The level of agreement on the perspective toward pandemic preparation between consultant and resident surgeons had similar ratings for each pandemic preparations statement. For example, nearly two-thirds of the surgeons strongly agreed that it is important for the department to conduct pre-event training for infection disease outbreaks. Approximately 40% of the surgeons strongly agreed that the department should provide adequate training and preparation regarding pandemic response. Similarly, nearly half of the surgeons strongly agreed that they received information on Personal Protective Equipment (PPE) usage, viral testing, and self-quarantine guidelines. Likewise, 34% and 44.4% of the consultants and residents, respectively, strongly agreed that they received training for donning and doffing PPE, whereas 40.4% and 39.3% of consultants and residents strongly agreed that they were well prepared to respond to the pandemic and around 40% of each group strongly agreed that they feel safe in the work caring for patients during the pandemic.

### Surgeons’ response to the pandemic

Regarding surgeons’ response to the pandemic, 53.2% and 50% of consultants and residents strongly agreed that consultant physicians share an obligation in planning for response and recovery efforts during pandemic (p = 0.552) which includes resident physicians (p = 0.525). In addition, 44.7% and 48% of consultants and residents strongly agreed that they are willing to respond to the pandemic regardless of severity (p = 0.664). Moreover, when asked what capacity they’d be willing to respond, 42.6% of consultants indicated that only if the primary responsibilities were providing surgical services while 33.7% of the residents indicated at any capacity including non-medical capacities (p = 0.643).

### Surgeons’ perspectives on how surgical training was impacted during the pandemic

Regarding the impact of surgical training during the pandemic, this has been measured by 5-item questionnaires with 5-point Likert scale categories where the answer option ranging from strongly agree to strongly disagree. Statistical analysis revealed that 40.4% and 32.7% of consultants and residents, respectively reported that the residency program was prepared and transitioned to a virtual curriculum (p = 0.260). Approximately, 22% and 27% of consultants and residents strongly agreed that there was a similar curriculum between virtual and in-person training (p = 0.548). Furthermore, more than 70% of consultants and residents agreed or strongly agreed that the development of clinical skills in both inpatient and outpatients will be negatively affected this year (p = 0.668) and approximately 40% of the surgeons strongly agreed that the procedural or operative skills of residents will also be negatively affected due to the pandemic (p = 0.908). (Table 2)

**Table 2:** Surgeons’ perspectives on how surgical training was impacted this year during the pandemic ^(n=243)^

Statement	Consultants N (%) (n = 47)	Residents N (%) (n = 196)	P-value ^§^
The residency program was prepared and transitioned well to a virtual curriculum			
• Strongly agree	19 (40.4%)	64 (32.7%)	
• Agree	11 (23.4%)	61 (31.1%)	
• Neutral	08 (17.0%)	51 (26.0%)	0.260
• Disagree	07 (14.9%)	15 (07.7%)	
• Strongly disagree	02 (04.3%)	05 (02.6%)	
The virtual curriculum will train residents as well as the in-person curriculum			
• Strongly agree	10 (21.3%)	52 (26.5%)	
• Agree	12 (25.5%)	52 (26.5%)	
• Neutral	10 (21.3%)	52 (26.5%)	0.548
• Disagree	10 (21.3%)	27 (13.8%)	
• Strongly disagree	05 (10.6%)	13 (06.6%)	
I am concerned the residents will not be as well trained or fall behind in their overall training this year due to the pandemic			
• Strongly agree	15 (31.9%)	58 (29.6%)	
• Agree	18 (38.3%)	82 (41.8%)	
• Neutral	09 (19.1%)	35 (17.9%)	0.990
• Disagree	03 (06.4%)	14 (07.1%)	
• Strongly disagree	02 (04.3%)	07 (03.6%)	
I am concerned that the development of the residents’ clinical skills, both inpatient and outpatient, will be impaired this year			
• Strongly agree	17 (36.2%)	76 (38.8%)	
• Agree	16 (34.0%)	73 (37.2%)	
• Neutral	11 (23.4%)	32 (16.3%)	0.668
• Disagree	01 (02.1%)	10 (05.1%)	
• Strongly disagree	02 (04.3%)	05 (02.6%)	
I am concerned that the procedural or operative skills of residents will be adversely affected this year			
• Strongly agree	18 (38.3%)	75 (38.3%)	
• Agree	15 (31.9%)	69 (35.2%)	
• Neutral	09 (19.1%)	36 (18.4%)	0.908
• Disagree	04 (08.5%)	10 (05.1%)	
• Strongly disagree	01 (02.1%)	06 (03.1%)	

^§^ P-value has been calculated using Chi-square test.

### Surgeons’ perspectives on disaster medicine training

Pertaining to the perspectives on disaster medicine training, 53.2% and 43.4% of the consultant and resident surgeons reported having previous experience in disaster medicine training (p = 0.224). Nearly 80% in each group believed that the program should incorporate routine disaster medicine (p = 0.804). Of those who answered “yes”, 60.9% of the residents believe that it should be included in the curriculum while 54.1% of the consultants believe that it should be a part of continuing medical education requirements (p = 0.098). Similarly, 46.8% and 47.4% of the consultants and residents indicated that the suitable frequency of disaster medicine training was every year (p = 0.083). In addition, 80.9% and 77% of the consultants and residents that the best method for disaster medicine training (p = 0.573) is simulation, which is a learning method allowing for skill acquisition via practice rather than mere observation. (Table 3)

**Table 3:** Surgeons’ perspectives on disaster medicine training ^(n=243)^

Statement	Consultants N (%) (n = 47)	Residents N (%) (n = 196)	P-value ^§^
Do you have any experience in disaster medicine?			
• Yes	25 (53.2%)	85 (43.4%)	0.224
• No	22 (46.8%)	111 (56.6%)	
In your opinion, should the program incorporate routine disaster medicine training?			
• Yes	37 (78.7%)	151 (77.0%)	0.804
• No	10 (21.3%)	45 (23.0%)	
If you answered with “yes” in the previous question, which form of incorporation should be implemented			
• It should be included into the training curriculum	17 (45.9%)	92 (60.9%)	
			0.098
• It should be a part of continuing medical education requirements			
	20 (54.1%)	59 (39.1%)	
The suitable frequency of disaster medicine training			
• Every year	22 (46.8%)	93 (47.4%)	
• Every 6 months	12 (25.5%)	28 (14.3%)	0.083
• Every 3 months	02 (04.3%)	31 (15.8%)	
• At orientation	11 (23.4%)	44 (22.4%)	
The suitable method of disaster medicine training			
• Simulation	38 (80.9%)	151 (77.0%)	0.573
• Lecture	09 (19.1%)	45 (23.0%)	

^§^ P-value has been calculated using Chi-square test

**Fig. 2:**
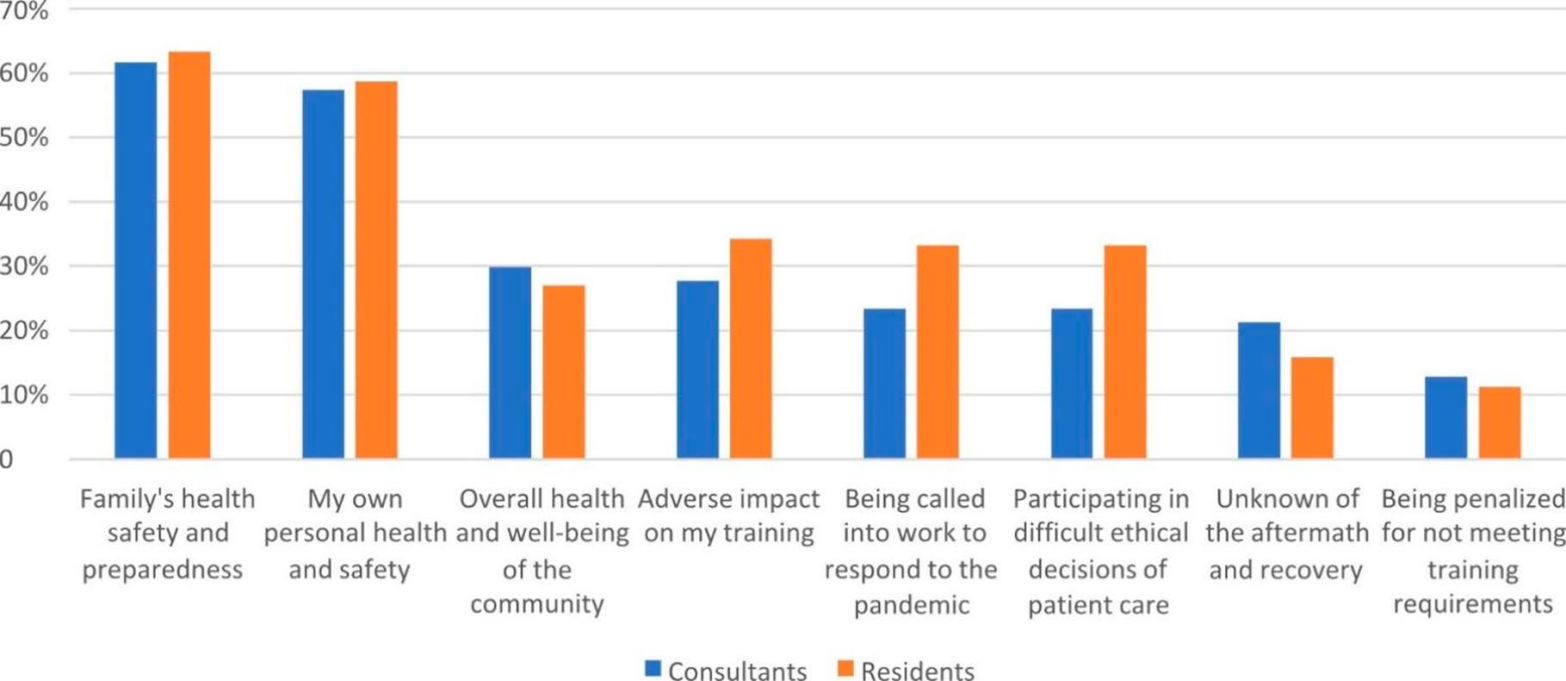
Concerns of Surgeons during the COVID-19 pandemic

In Fig. 2, the most common concerns of consultant surgeons during the COVID-19 pandemic were family’s health, safety, and preparedness, followed by the personal health and safety and overall health and well-being of the community while for resident surgeons, the most common concerns were family’s health, safety, and preparedness, personal health and safety and adverse impact of the pandemic to residency training.

**Fig. 3:**
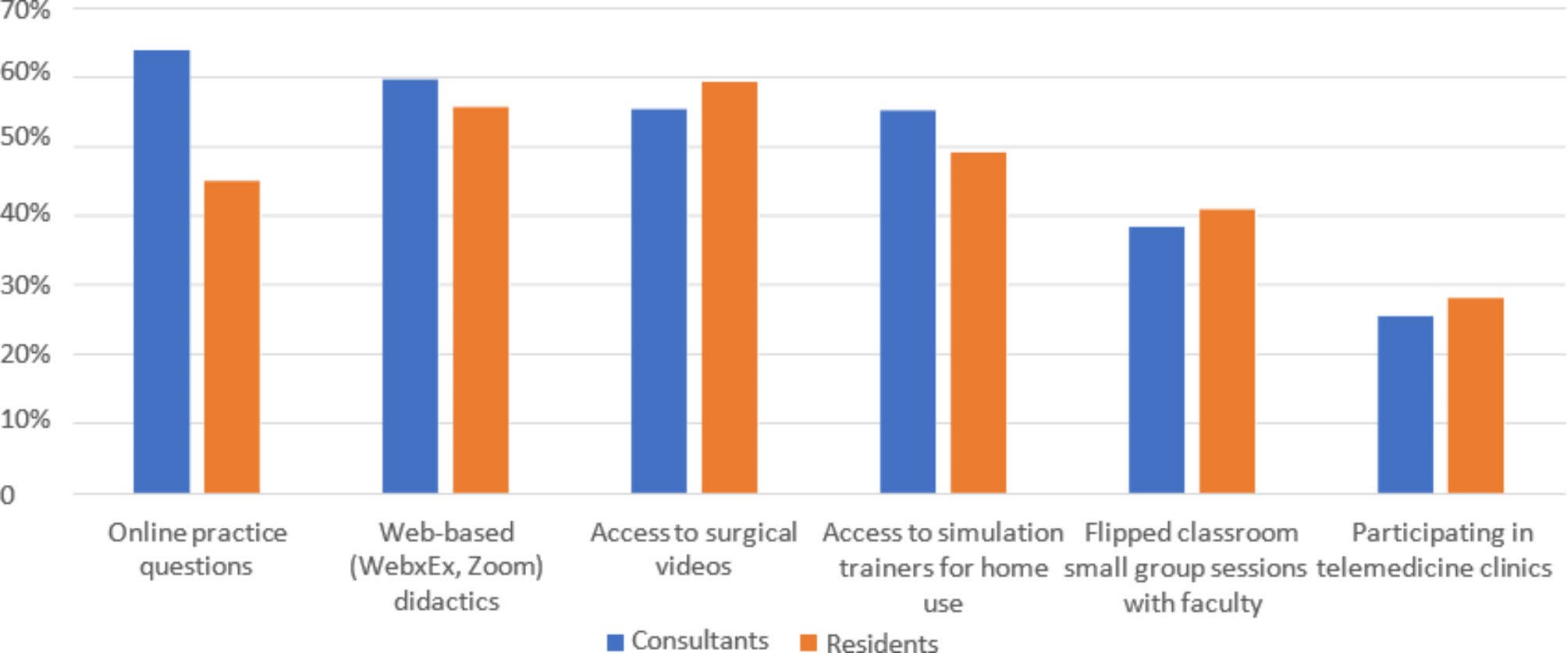
Surgeons’ requisite virtual curriculum components

In Fig. 3, the most common prerequisite of consultant surgeons about virtual curriculum components was online practice questions, followed by web-based and access to surgical videos, while resident surgeons indicated access to surgical videos, followed by web-based and access to simulation trainers for home use.

## Discussion

On a global scale, the negative impact of the COVID-19 pandemic was on several elements of healthcare. Unfortunately, patients’ mortality as a direct result of the virus and the effect on their relatives and close friends remains the pandemic’s primary and most profound loss. This has influenced the international healthcare workforce’s health and well-being. [11] The expected number of elective surgery cancellations is unprecedented [2], necessitating modifications in service delivery and staffing to continue providing good care. Prior to the pandemic, there were deficiencies in surgical training. Mandatory training experience surveys have shown areas of distress, such as gaps in the rota, absence of protected personal reading time, and meeting theatrical quality standards. [11] The pandemic has exacerbated the situation. According to the 2020 General Medical Council nationwide questionnaire [12], 80% of doctors in training responded that coronavirus interruption limited their access to the information they required to advance in their field. Importantly, the findings from our study are consistent with these findings. To assess the impact of the COVID-19 pandemic on all specialties’ national basis, surgical training was the main aim of this study, which is the first of its kind in Saudi Arabia. The COVID-19 epidemic has clearly had a significant impact on surgical education and training. The long-term impact of the COVID-19 pandemic on surgical education in Saudi Arabia, on the other hand, has received little attention in the literature. This study aimed to assess surgical residents’ and consultants’ impressions of COVID-19 impact on their education in Saudi Arabia and investigate the gaps that emerged in several parts of local surgical training.

The Saudi Commission of Health Specialties (SCHS) is the governing body that is responsible for the supervision and evaluation of the local training programs and setting the standards for healthcare professionals’ practice. The SCHS’s residency programs for general surgery do not require disaster medicine [13], which includes training for infectious disease pandemics, as it does for emergency medicine. [14] As a result, surgical residents receive far little, if any, disaster medicine training. [15, 16] Surgical consultants also lack disaster medicine experience, with about 53.2% of surgeons having any previous experience in disaster medicine. Consequently, the importance of disaster medicine may have been missed prior to the pandemic. However, as indicated in our study, our residents and consultants increasingly recognize the significance of including routine disaster medicine training in the curriculum and continuing medical education. The most suitable method for disaster medicine training was the use of simulation. Before the pandemic, national platforms stressed the health and well-being of surgical residents and consultants. According to a nationwide study of general surgery residents, 38.5% had weekly stress and burnout features, with 4.5% expressing suicide ideation. [17] The American College of Surgeons conducted a poll of approximately 8000 surgeons and found a 53% prevalence of burnout, much higher than the general population average of 28%. [18] As seen by a poll of Wuhan healthcare professionals who reported instances of 50% sadness, 44% anxiety, 34% insomnia, and 71% distress [19], the pandemic has only exacerbated these psychological issues. Hence in this study, our surgical residents’ main concern was their families’ health, safety, and preparation. Being more thoughtful in providing the residents time away from work during the daytime hours to take care of family chores might be one strategy to address the well-being of families, mainly because community services have restricted operating hours during the pandemic. When testing, immunization, and treatment are available, programs may need to assess the practicality of prioritizing the resident’s families.

Most residents (63.8%) thought e-learning opportunities taught them just as effectively as the in-person lectures, while most consultants disagreed. This disparity in perception might be attributable to age disparities and residents’ and consultants’ exposure to technology. This might also explain why consultants might prefer the more traditional prerequisites such as online practice questions rather than simulation software and videos, which residents prefer. The generational gaps need to be taken into account when discussing e-learning and adopting newer educational plans. These findings are consistent with a study conducted to explore the opinions of Saudi plastic surgery consultants and residents on the implication of e-learning during the COVID-19 pandemic. [20] Hybrid or blended learning, which combines conventional and virtual teaching, might be the way of the future in surgical education. While our residents may be comfortable with web-based systems, our consultants may not be. [21] Web-based videos, access to surgical videos, and access to simulation trainers for home use were the most prerequisite for both residents and consultants. However, consultants prefer online practice questions more than the residents. As a result, the time restrictions of surgical faculty and the scale of clinical demands remain unknown as we continue to teach residents in a pandemic context. Maintaining a consistent, resilient, and intense training experience requires a multi-institutional didactic education strategy.

To equip the next generation of consultants in surgery with the abilities to provide safe and effective patient care, high-quality training is required. As a result, the COVID-19 pandemic recovery phase should be seen as a chance to introduce and build a new, better standard, not only to restore regular surgical services and training. Although the actual effect and implications of the pandemic will not be known for some time, our research has shown that surgical consultants and residents viewed that surgical education and training in all surgical specialties in Saudi Arabia has been negatively affected. Our findings are consistent with a study conducted by Balhareth et al. They found that 84.6% of their respondents reported a reduction in training activities. Approximately 97% of those with surgical specialties noted a reduction in their surgical exposure due to the pandemic. [22]

## Limitations and recommendations

Even though the study’s objectives have been achieved, several limitations must be mentioned. The main strength of this study was that it was a nationwide cross-sectional study that included participants from all over Saudi Arabia. Another strength is that this study was conducted after a year and a half since the declaration of the pandemic, so we were able to inquire about late views of surgical residents and consultants, which might give accurate feedback on the changes made by the pandemic on the local training. The study’s cross-sectional design may be prone to recollection bias, question misunderstanding, and reporter bias, which is the main limitation. Also, the generalization of this study’s results is limited as the tool used for data collection was an online survey, so respondents were only those with access to online services. Another major limitation may be the residents’ perspectives, as they may not fully know what is relevant for their surgical training in the future; they possess a relatively limited perspective. The timing of data collection was approximately 7 months after the approval of the first COVID-19 vaccine (Pfizer-BioNTech). So, the authors could not explore the possibility of different answers before and after vaccination approval. Finally, the specific needs regarding the surgical training can differ from one specialty to another, and given the nature of the study design, defining these exact needs would be not possible. Thus, further future comparative qualitative studies are highly recommended. Despite these limitations, we provide the perceptions of both surgical residents and consultants regarding how to improve the surgical training and make it more resilient to such pandemics.

## Conclusion

COVID-19 pandemic has obviously impacted the educational environment of surgical training across the world. Importantly, it has taught us that former means of education and learning have to be adapted to the new changes. Addressing the concerns of both residents and consultants followed by implementing new components to the surgical education is critical. Thus, we recommend adequately exposing residents and consultants to disaster medicine training, improving the virtual curriculum, enhancing their well-being by offering psychological consultations, and alleviating their concerns over their families’ safety by prioritizing their access to immunization and treatment.

## Electronic supplementary material

Below is the link to the electronic supplementary material.

Supplementary Material 1

## Data Availability

The datasets generated and/or analyzed during the current study are not publicly available due to respondents’ confidentiality but are available from the corresponding author on reasonable request.
