# Supplementary material for: The lasting impact of COVID-19 on surgical training from the perspective of surgical residents and consultants in Saudi Arabia: a nationwide cross-sectional study
Source: BMC Med Educ. 2023 May 11;23:330. doi: 10.1186/s12909-023-04302-4 (PMC10173899; doi:10.1186/s12909-023-04302-4)
Supplement: Supplementary file 1 — Supplementary Material 1 [file 12909_2023_4302_MOESM1_ESM.pdf]

The Lasting Impact of COVID-19 on Surgical Training from the Perspective of Surgical Residents and Consultants in Saudi Arabia: A Nationwide Cross-Sectional Study

* Required

1. Do you agree to participate? *

Mark only one oval.

- Agree
- Disagree

2. You are: *

Mark only one oval.

- Resident *Skip to question 3*
- Attending/Consultant *Skip to question 4*

..

3. Level of residency: *

Mark only one oval.

- R1
- R2
- R3
- R4
- R5
- R6
- R7

Demographics

4. Age: *

Mark only one oval.

- ( ) Under 25 years old
- ( ) 25–35 years old
- ( ) Above 35 years old

5. Gender: *

Mark only one oval.

- ( ) Female
- ( ) Male

6. Nationality: *

Mark only one oval.

- ( ) Saudi
- ( ) Others

7. If you chose "others" in the previous question, please specify your nationality:

____

8. Marital status: *

Mark only one oval.

- ( ) Single
- ( ) Married
- ( ) Divorced
- ( ) Widowed
- ( ) Prefer not to answer

9. You live in: *

Mark only one oval.

- Northern region of Saudi Arabia
- Central region of Saudi Arabia
- Eastern region of Saudi Arabia
- Western region of Saudi Arabia
- Southern region of Saudi Arabia

10. You work in: *

Mark only one oval.

- Tertiary Hospital
- Secondary Hospital
- Primary Hospital

11. Speciality: *

Mark only one oval.

- Surgical
- Non-surgical

12. If your speciality is surgical, choose one of the following:

Mark only one oval.

- Trauma/Acute Care
- Cardiothoracic
- Plastic Surgery
- Neurosurgery
- General Surgery
- Obstetrics and gynaecology
- Orthopedics
- Ear nose and throat surgery
- Ophthalmology
- others

13. If your speciality is non-surgical, choose one of the following:

Mark only one oval.

- Internal Medicine
- Emergency Medicine
- Preventive Medicine
- Family Medicine
- Community Medicine
- Anesthesiology
- Radiology
- Pathology
- Dermatology
- Others

14. Years of Experience working as a physician: *

Mark only one oval.

- ≤5 years
- 6–10 years
- >10 years

The questionnaire

Please answer the following questions aacording to your perspective during COVID-19 pandemic:

15. Pandemic Preparation: *

Mark only one oval per row.

	Strongly agree	Agree	Neutral	Disagree	Strongly disagree
**It is important for the department to provide pre-event training for infectious disease outbreaks**	⬭	⬭	⬭	⬭	⬭
**The department provided adequate preparation and training for infectious disease outbreaks**	⬭	⬭	⬭	⬭	⬭
**I received information on PPE usage, viral testing, and self-quarantine guidelines**	⬭	⬭	⬭	⬭	⬭
**I received training for donning and doffing PPE including the PAPR**	⬭	⬭	⬭	⬭	⬭
**I feel sufficiently prepared to respond to the pandemic**	⬭	⬭	⬭	⬭	⬭
**I am safe in my work caring for patients during the pandemic**	⬭	⬭	⬭	⬭	⬭

16. Do you have any experience in disaster medicine? *

Mark only one oval.

- ⬭ Yes
- ⬭ No

17. In your opinion, shoud the program incorporate routine disaster medicine training? *

Mark only one oval.

- ⬭ Yes
- ⬭ No

18. If you answered with "yes" in the previous question, which form of incorporation should be implemented:

Mark only one oval.

- It should be included into the training curriculum.
- It should be a part of continuing medical education requirements.

19. The suitable frequency of disaster medicine training: *

Mark only one oval.

- Every year
- Every 6 months
- Every 3 months
- At orientation

20. The suitable method of disaster medicine training: *

Mark only one oval.

- Simulation
- Lecture

21. Clinical response to the pandemic: *

Mark only one oval per row.

	Strongly agree	Agree	Neutral	Disagree	Strongly disagree
**Attending physicians share an obligation in the planning, response and recovery efforts during the pandemic**					
**Resident physicians share an obligation in the planning, response and recovery efforts during the pandemic**					
**I would be willing to respond to the COVID-19 pandemic regardless of severity**					

22. What capacity would you be willing to respond in the current pandemic? *

Mark only one oval.

- Any capacity including non-medical capacities.
- Any medical capacity including re-deployment to other specialty teams.
- Only if my primary responsibilities were providing surgical services.
- If I were given the option, I would prefer to stay home and not respond.

23. Please answer the two following questions considering the psychological preparedness to face COVID-19 pandemic: *

Mark only one oval per row.

	Yes	No
**Do you have an access to psychological support and counseling?**		
**Are you psychologically prepared to respond to the pandemic?**		

24. Please rank the following concerns during COVID-19 pandemic in order of importance: *

Mark only one oval per row.

	Family's health, safety, and preparedness	My own personal health and safety	Adverse impact on my training	Overall health and well-being of the community	Being penalized for not meeting training requirements	Unknown of the aftermath and recovery	Being called in to work to respond to the pandemic	Participating in difficult ethical decisions of patient care
**1st choice**	⬭	⬭	⬭	⬭	⬭	⬭	⬭	⬭
**2nd choice**	⬭	⬭	⬭	⬭	⬭	⬭	⬭	⬭
**3rd choice**	⬭	⬭	⬭	⬭	⬭	⬭	⬭	⬭
**4th choice**	⬭	⬭	⬭	⬭	⬭	⬭	⬭	⬭
**5th choice**	⬭	⬭	⬭	⬭	⬭	⬭	⬭	⬭
**6th choice**	⬭	⬭	⬭	⬭	⬭	⬭	⬭	⬭
**7th choice**	⬭	⬭	⬭	⬭	⬭	⬭	⬭	⬭
**8th choice**	⬭	⬭	⬭	⬭	⬭	⬭	⬭	⬭

25. Impact of COVID-19 on the training: *

Mark only one oval per row.

	Strongly agree	Agree	Neutral	Disagree	Strongly disagree
**The residency program was prepared and transitioned well to a virtual curriculum**	⬭	⬭	⬭	⬭	⬭
**The virtual curriculum will train residents as well as the in-person curriculum**	⬭	⬭	⬭	⬭	⬭
**I am concerned the residents will not be as well trained or fall behind in their overall training this year due to the pandemic**	⬭	⬭	⬭	⬭	⬭
**I am concerned that the development of the residents' clinical skills, both inpatient and outpatient, will be impaired this year**	⬭	⬭	⬭	⬭	⬭
**I am concerned that the procedural or operative skills of residents will be adversely affected this year**	⬭	⬭	⬭	⬭	⬭

26. Please identify the most essential components of a virtual curriculum: *

Check all that apply.

- [ ] Web-based (WebxEx, Microsoft Teams, Zoom) educational programs
- [ ] Access to surgical/medical videos
- [ ] On-line practice questions
- [ ] Access to simulation trainers for home use
- [ ] Flipped classroom small group sessions with faculty
- [ ] Participating in tele-medicine clinics

This content is neither created nor endorsed by Google.

Google Forms
